# Precursor-Dependent Initial Coulombic Efficiency of Hard Carbon Anodes for Sodium-Ion Batteries: A Comparative Review

**DOI:** 10.3390/ma19102132

**Published:** 2026-05-19

**Authors:** Xuchen Huang, Zhiyi Wang

**Affiliations:** 1 Sino-German College of Engineering, Qingdao University of Science and Technology, No. 6 Xiaoqinghe Road, Huangdao District, Qingdao 266042, China; 2 College of Materials Science and Engineering, Qingdao University of Science and Technology, No. 53 Zhengzhou Road, Qingdao 266042, China

**Keywords:** hard carbon, sodium-ion batteries, initial Coulombic efficiency, precursor selection, closed pore

## Abstract

Hard carbon has been widely recognized as the most commercially viable anode material for sodium-ion batteries (SIBs); however, its inherently low initial Coulombic efficiency (ICE), typically 60–90%, remains a critical bottleneck constraining practical full-cell deployment. While extensive research has addressed ICE optimization, existing reviews have predominantly focused on individual precursor types or isolated strategies, lacking a unified cross-precursor comparative framework. This review systematically deconstructs the complete causal continua—from chemical composition through carbonization trajectories and microstructural evolution to ultimate ICE outcomes—across five major precursor categories: biomass, synthetic resins, pitches, coal-based materials, and saccharides. An “SSA-closed pore–defect” three-parameter trade-off framework is proposed to elucidate the microstructural origins of precursor-dependent ICE divergences. Cross-categorical benchmarking reveals that resin-based precursors achieve the highest ICE (95%) through ultra-low specific surface area and extensive closed porosity, pitch-based systems deliver the most consistent ICE distribution (86–91%), and coal-derived carbons are confined to the lowest tier (78–85%). The differentiated efficacy of carbonization conditions and post-treatment strategies across precursor types is critically evaluated, demonstrating that optimal process selection is inextricably linked to precursor taxonomy. Building upon these analyses, a precursor selection decision roadmap targeting three application-specific ICE thresholds is constructed, providing actionable guidance for matching precursor–process combinations to industrial requirements. The comparative framework is grounded in 25 representative studies selected through explicit inclusion criteria (detailed in the Introduction), and its predictive utility is illustrated for emerging precursor candidates beyond the five canonical categories. This cross-precursor perspective offers a systematic reference for accelerating the commercialization of hard carbon anodes in SIBs.

## 1. Introduction

Sodium-ion batteries (SIBs) have emerged as a promising complementary technology to lithium-ion batteries for large-scale energy storage, owing to sodium’s natural abundance, cost-effectiveness, and intrinsic safety advantages [1,2]. Among candidate anode materials, hard carbon is currently the only commercially viable option for SIBs, attributed to its disordered turbostratic structure, moderate interlayer d-spacing, and strong sodium-ion storage capability [3,4]. However, the initial Coulombic efficiency (ICE) of hard carbon anodes is typically constrained to 60–90%, representing a critical bottleneck for practical full-cell deployment.

ICE is defined as the ratio of the first desodiation capacity to the initial sodiation capacity [5]. Because the sodium inventory in a full cell is exclusively cathode-derived, any irreversible first-cycle loss at the anode—primarily through SEI formation, defect-site trapping, and surface oxygen reactions—directly translates into diminished full-cell energy density and shortened cycle life [6,7]. Qin et al. [8] quantified this effect: raising hard carbon ICE from 75% to 90% yields approximately 20% enhancement in full-cell energy density, underscoring ICE optimization as the paramount challenge in scaling hard carbon anodes to industrial deployment.

Extensive research has targeted ICE optimization, and several recent reviews have synthesized these advances from complementary perspectives: microstructural engineering [9], surface chemistry [10], sodium storage mechanisms [11], sustainable precursor selection [12], scalable manufacturing [13], and synthesis–property–performance correlations [14,15]. Beyond hard-carbon batteries, pioneering work on biomass-derived electrodes for sodium energy devices—including aqueous Na systems and Na-ion hybrid capacitors—by Minakshi and co-workers has established the broader relevance of biomass carbons in Na energy storage [16]. Despite these contributions, the following three fundamental gaps persist: (1) the absence of a unified, precursor-centric framework that benchmarks ICE across the five primary precursor categories—biomass, synthetic resins, pitches, coal, and saccharides/simple organics; (2) the lack of a cohesive causal-chain analysis from chemical composition through carbonization to microstructure and ICE across these categories; and (3) the paucity of full-cell-oriented decision guides targeting specific ICE thresholds.

To bridge these gaps, this review offers three complementary perspectives. First, a precursor-resolved ICE–capacity dataset is compiled from 25 representative studies and cross-compared, with selection criteria detailed below. Second, the complete causal chains (“composition → carbonization → microstructure → ICE”) for all five precursor categories are delineated in parallel, elucidating the precursor-dependent divergence in ICE regulation mechanisms. Third, a precursor selection decision roadmap targeting specific ICE thresholds is proposed to provide actionable guidance for industrial precursor design. By transcending the single-precursor or isolated-strategy paradigms prevalent in existing reviews, this comparative framework aims to accelerate the commercialization of hard carbon anodes in SIBs [17,18].

To ensure transparency in the cross-precursor benchmarking, Table 1 compiles 25 representative studies retrieved from Web of Science and Scopus (2015–2025) using combinations of the keywords “hard carbon”, “sodium-ion battery”, “initial Coulombic efficiency”, and precursor-specific terms; foundational pre-2015 works are additionally cited where historically indispensable. Studies were retained if they satisfied all of the following: reported ICE together with at least three of the four core structural descriptors (SSA, d_002_, closed pore volume, I_D/I_G); employed half-cell testing at current densities of 20–50 mA g^−1^ over a voltage window of 0–2.0 V (minor deviations are footnoted in Table 1); and used a hard-carbon single-phase working electrode. Five studies were retained for each precursor category to span the reported ICE range while prioritizing characterization completeness, constituting a representative rather than exhaustive sampling. No numerical normalization across testing conditions was imposed—such heterogeneity itself is a documented source of ICE dispersion [9]—and unreported parameters (most notably closed pore volume, quantitatively reported in only 2 of 25 entries) reflect the absence of a universally adopted measurement protocol [19,20,21] rather than selection bias; this limitation is addressed as a standardization priority in Section 6.

**Figure 1:**
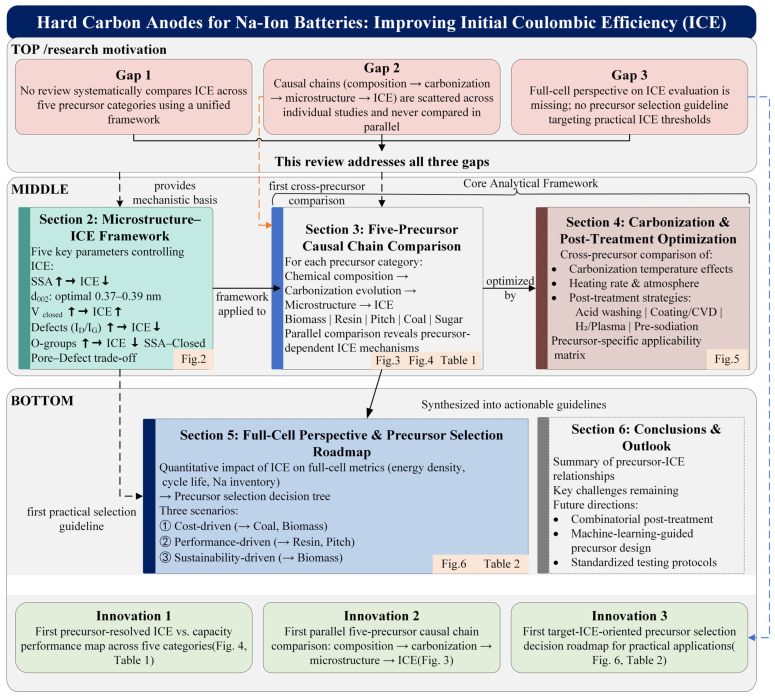
Content organization and logic flow of this Review. Arrows indicate the progression of the review from microstructural parameters and precursor categories to process optimization, full-cell relevance, and future perspec.

The structure of this review is organized as follows (Figure 1): Section 2 establishes the correlative framework between microstructural parameters and ICE, elucidating the individual impacts and interactive trade-offs among five critical variables—specific surface area, interlayer spacing, closed pore volume, defect concentration, and oxygen-containing functional groups. Section 3 constitutes the analytical core, providing in-depth causal chain analyses and cross-categorical benchmarking of hard carbons derived from the five distinct precursor families. Section 4 critically evaluates the differentiated effects of carbonization conditions and post-treatment strategies across different precursor types. Section 5 translates ICE metrics into full-cell performance parameters, culminating in the construction of the precursor selection decision roadmap. Finally, Section 6 summarizes the overarching conclusions and outlines pivotal directions for future investigation.

## 2. Correlative Framework Between Hard Carbon Microstructural Parameters and ICE

**Figure 2:**
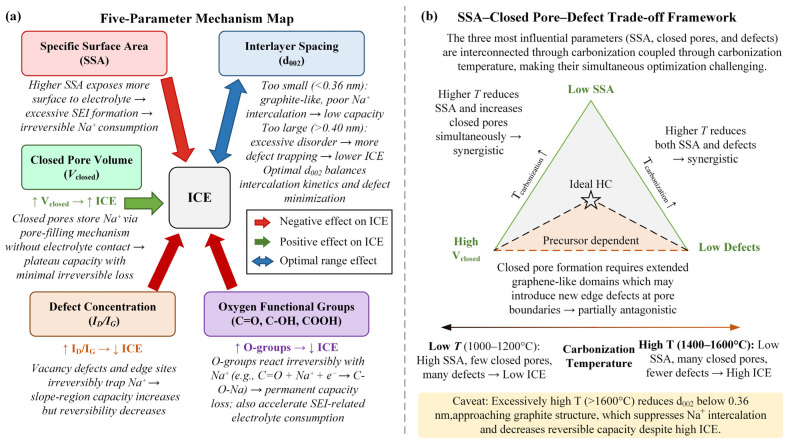
(**a**) Five-parameter mechanism map linking microstructural variables to ICE; green arrows indicate positive effects on ICE, whereas red arrows indicate negative effects on ICE (**b**) SSA–closed pore–defect trade-off framework as a function of carbonization temperature; arrows indicate the evolution trends of structural parameters with increasing carbonization temperature.

Prior to examining specific precursors, establishing a correlative framework between microstructural parameters and ICE is imperative. This framework provides a unified evaluation baseline for the subsequent causal chain analyses across the five precursor categories. This section delineates five critical structural parameters, elucidating their respective mechanistic impacts on ICE (Figure 2a), and culminates in a three-parameter trade-off analysis framework (Figure 2b).

### 2.1. Specific Surface Area (SSA)

Specific surface area (SSA) is a primary structural determinant of ICE. Beda et al. [46] systematically investigated the relationship between SSA and irreversible capacity across multiple hard carbons, showing that a 1 m^2^ g^−1^ increment in SSA corresponds to approximately 1–2 mAh g^−1^ of additional irreversible capacity—a linear correlation broadly applicable across precursor types. Mechanistically, higher SSA exposes more carbon surface to the electrolyte, inducing excessive solid electrolyte interphase (SEI) formation and irreversible electrolyte decomposition during the first cycle. Ghimbeu et al. [47] further deconvoluted the respective contributions of open pores and micropores, demonstrating through combined N_2_ and CO_2_ adsorption that the micropore fraction (<2 nm) exerts the most pronounced detrimental impact on ICE. This mechanism is directly reflected in the dataset compiled in Table 1, as follows: bamboo-derived hard carbon with SSA as low as 2.37 m^2^ g^−1^ [22] achieves an ICE of 93.9% with a reversible capacity of 324 mAh g^−1^, whereas red-yeast-rice-derived samples with SSA of 38.9 m^2^ g^−1^ [25] are limited to ~65% ICE despite comparable capacity. Attenuating SSA—particularly minimizing open microporosity—therefore constitutes a primary lever for ICE optimization.

### 2.2. Graphitic Interlayer Spacing (d_002_)

The d_002_ parameter describes the structural ordering within the turbostratic domains of hard carbon. The seminal work by Stevens and Dahn [48] established the intercalation–adsorption dual-mechanism model for sodium-ion storage, wherein d_002_ is a principal determinant of the intercalation capacity in the low-voltage plateau region. Bommier et al. [49] identified an optimal d_002_ window of approximately 0.37–0.39 nm. Excessively contracted d_002_ (<0.36 nm) drives carbon-layer stacking toward a graphitic configuration, thermodynamically restricting Na^+^ intercalation and diminishing both reversible capacity and ICE. Conversely, excessively expanded d_002_ (>0.40 nm) signifies a highly disordered architecture replete with defect sites that amplifies irreversible sodium trapping. The correlation between d_002_ and ICE is therefore non-monotonic, manifesting as an optimal-range effect that requires precise structural regulation. This optimal-range effect is directly reflected in the Table 1 dataset: pyrolyzed anthracite with d_002_ ≈ 0.360 nm [36] delivers only 222 mAh g^−1^ owing to constrained intercalation within a near-graphitic stacking, whereas HMTA-crosslinked phenolic resin within the optimal window (d_002_ = 0.381 nm, [26]) achieves 431 mAh g^−1^ with an ICE of 95.0%—demonstrating that the capacity–ICE benefit peaks precisely within the 0.37–0.39 nm regime identified above.

### 2.3. Closed Pore Volume

Closed pore engineering has emerged as a focal point in hard carbon research. Au et al. [50] refined the conventional sodium storage paradigm by proposing that sodium cluster accumulation within closed pores constitutes the dominant source of low-voltage plateau capacity. Subsequent investigations have further elucidated this mechanism, as follows: a 2025 study redefined closed pore classification criteria via solvation structure analysis [19]; Zhang et al. [51] isolated the discrete capacity contributions of ultramicropores versus closed pores; and Duan et al. [52] introduced the concept of semi-enclosed ultramicropores to enable rapid sodium storage kinetics. The positive influence of closed pores on ICE stems from their physical isolation from the electrolyte [20], as follows: sodium ions diffuse through carbon layers to populate these closed pores without initiating parasitic electrolyte decomposition, thereby delivering highly reversible plateau capacity. Quantitatively, Kamiyama et al. [44] demonstrated that MgO-templated closed pores in glucose-derived hard carbon deliver a record reversible capacity of 478 mAh g^−1^ and ICE of 88%, representing one of the highest combined capacity–ICE values reported to date. Guo et al. [21] proposed that a multimodal approach—combining true density measurement, small-angle X-ray scattering (SAXS), and transmission electron microscopy (TEM)—currently provides the most reliable protocol for closed pore quantification. Nevertheless, quantification methods remain non-standardized: true density, SAXS fitting, and TEM-based analyses often yield systematically different values, necessitating caution in cross-study comparisons. Only two entries in Table 1 report exact closed pore volumes ([23,26]), reflecting the prevailing difficulty in characterizing this parameter.

### 2.4. Defect Concentration (I_D/I_G)

The intensity ratio of the D to G bands (I_D/I_G) in Raman spectroscopy is the standard metric for quantifying defect concentration in hard carbons. Olsson et al. [53] used density functional theory (DFT) to map the spatial distribution of vacancies, edge defects, and Stone–Wales defects, and their differential affinities for alkali metal storage. Sun et al. [54] empirically demonstrated that residual oxygen heteroatoms and vacancy defects induce a “trapping effect,” in which sodium ions are irreversibly sequestered at these sites—augmenting sloping-region capacity but penalizing ICE. Li et al. [55] provided direct evidence for the defect–capacity correlation: microwave-treating a cellulose-derived hard carbon for 6 s preserves high defect density and raises reversible capacity from 204 to 308 mAh g^−1^, substantially exceeding the 274 mAh g^−1^ delivered by the same precursor annealed at 1100 °C for 7 h, which depletes defect sites. The quasi-linear relationship between I_D/I_G and sloping capacity has been independently established by Bommier et al. [56] across turbostratic carbons spanning a wide range of domain sizes. This defect-driven capacity gain, however, comes at the cost of ICE, as follows: Table 1 reveals that the lowest-defect sucrose hard carbon [43] (I_D/I_G ~ 0.8) achieves ICE of 86.1% with 361 mAh g^−1^ reversible capacity—i.e., high ICE at the expense of total capacity—whereas high-defect biomass samples typically fall below 75% ICE [24,25]. Huang et al. [57] further cautioned that indiscriminate defect elimination precipitates a concomitant loss of sloping capacity, underscoring the need for balanced defect engineering rather than defect maximization or minimization.

### 2.5. Oxygen-Containing Functional Groups

Surface oxygen functionalities (e.g., C=O, C–OH, COOH) represent another primary vector for irreversible sodium consumption. Using cellulose-derived hard carbon as a model system, Zhao et al. [58] systematically modulated the speciation and concentration of these groups, showing that carbonyl (C=O) moieties undergo irreversible faradaic reactions with sodium ions (C=O + Na^+^ + e^−^ → C–O–Na) and directly amplify first-cycle irreversible capacity. Xu et al. [59] corroborated these findings in bamboo-derived architectures, demonstrating that surface oxygen depletion synergistically couples with pore-structure optimization to elevate ICE. This coupling is evident in the Table 1 dataset, as follows: bamboo hard carbon cross-linked at 1500 °C to eliminate oxygen functionalities [22] reaches ICE of 93.9%, whereas oxygen-rich biomass samples carbonized at lower temperatures (1300 °C, [24]) are confined to ICE below 72%. Crucially, the detrimental impact of oxygen functionalities is intimately coupled with SSA, as follows: expanded surface area inherently harbors a higher density of exposed oxygen groups, and these coupled variables synergistically exacerbate first-cycle irreversible losses.

### 2.6. SSA–Closed Pore–Defect Trade-Off Analysis Framework

Before presenting the trade-off framework itself, we briefly clarify why these three parameters—SSA, closed pore volume, and defect concentration—were selected as the framework axes. Comprehensive structure–property analyses by Dou et al. [60] and Matei Ghimbeu et al. [15] converge on the conclusion that ICE in hard carbons is primarily governed by the following three independent physical processes: (i) electrolyte decomposition at the electrolyte-accessible surface (captured by SSA), (ii) reversible Na-cluster accommodation in electrolyte-inaccessible voids (captured by closed pore volume), and (iii) irreversible Na trapping at undercoordinated carbon sites (captured by defect concentration). Other frequently invoked descriptors—heteroatom chemistry, true density, and pore size distribution—are not independent axes but derivatives of these three. Heteroatom effects, particularly those of oxygen groups, operate through SEI formation and defect-site interactions that are already captured by SSA and defect density (Section 2.5). True density is a mathematical proxy for closed pore volume; Jian et al. [61] explicitly formulated V_closed = 1/ρ_skeletal − 1/ρ_true using graphite skeletal density as the reference, demonstrating that true density measurements are used to calculate, not supplement, closed pore volume. Pore size distribution is itself extracted from the N_2_/CO_2_ adsorption isotherms used to derive SSA, and its electrochemically relevant fraction—ultramicropores below 2 nm—overlaps substantially with the closed-pore regime. The three-parameter framework therefore captures the minimum sufficient set of orthogonal variables controlling ICE.

The aforementioned five parameters do not operate independently; rather, they are coupled through carbonization temperature, the central thermochemical variable. Chen et al. [62] showed that elevating the carbonization temperature concurrently attenuates SSA, expands closed pore volume, and mitigates structural defects, yielding a synergistic enhancement of ICE. However, at extreme temperatures (>1600 °C), d_002_ contracts toward graphitic dimensions (<0.36 nm), which kinetically and thermodynamically impedes Na^+^ intercalation. The Raman framework of Ferrari and Robertson [63] underpins the non-monotonic evolution of I_D/I_G with elevated temperatures. More recently, Guo et al. [64] reported that closed pore expansion is occasionally accompanied by new edge defects along pore walls, revealing a partial mechanistic antagonism between closed pore development and defect annihilation.

Synthesizing these insights, we propose the “SSA–closed pore–defect” three-parameter trade-off framework (Figure 2b). Within this construct, concurrent SSA reduction and closed pore expansion are synergistically driven by rising carbonization temperature; similarly, SSA reduction and defect annihilation are cooperative. However, closed pore maximization and defect minimization exhibit a precursor-dependent antagonistic dynamic. This framework is applied in Section 3 to explain why different precursor families occupy divergent ICE regimes—each precursor’s unique thermochemical evolution dictates a distinct position within the three-parameter trade-off space.

## 3. Comparative ICE Analysis and Causal Chain Delineation Across Five Precursor-Derived Hard Carbons

**Figure 3:**
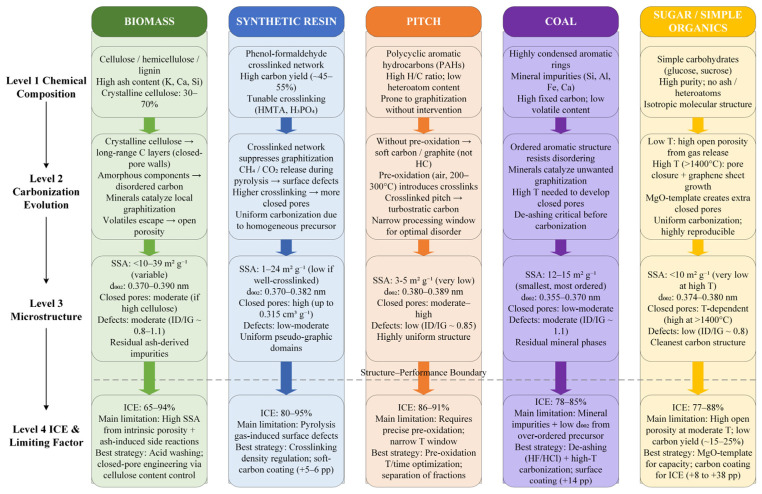
Comparative causal continua from precursor composition to ICE across five precursor categories.

**Figure 4:**
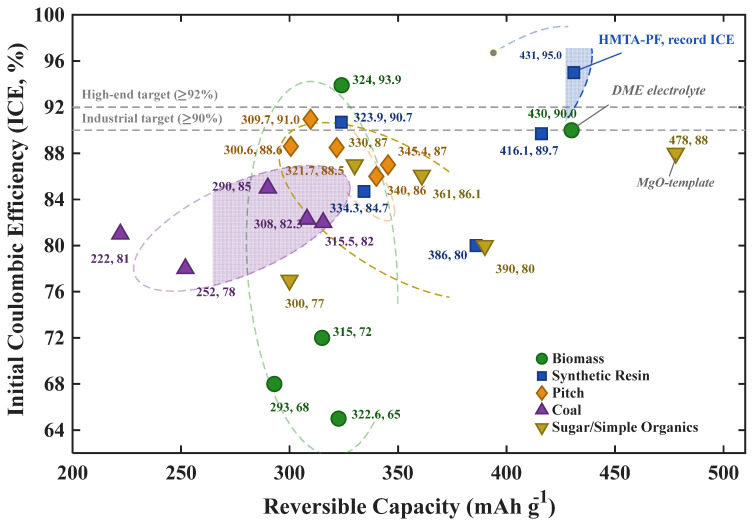
Scatter plot of ICE versus reversible capacity for hard carbons derived from five precursor categories. The data point measured in a DME-based ether electrolyte is annotated.

Building upon the microstructural parameter–ICE correlative framework established in Section 2, this section utilizes precursor taxonomy as the primary classification axis to systematically deconstruct the complete causal continua—from initial chemical composition through carbonization trajectories and microstructural evolution, to ultimate ICE outcomes—across five distinct precursor families: biomass, synthetic resins, pitches, coal-derived materials, and saccharides/low-molecular-weight organics (Figure 3). Cross-precursor benchmarking is quantitatively facilitated through an ICE–capacity scatter matrix (Figure 4) and a comprehensive data synthesis (Table 1). This section focuses exclusively on the structural and performance divergences originating from the intrinsic physicochemical characteristics of each precursor; the synergistic optimization of carbonization protocols and post-treatment engineering will be addressed holistically in Section 4.

### 3.1. Biomass-Derived Precursors

Biomass is the most abundant and economically accessible class of hard carbon precursors, encompassing wood, agricultural residues, fruit shells, and chitin. Its macromolecular composition is governed by varying ratios of cellulose, hemicellulose, and lignin, accompanied by relatively high inorganic ash fractions (e.g., K, Ca, Si) [22]. This compositional heterogeneity makes biomass-derived hard carbons exhibit the broadest ICE dispersion among the five precursor categories (65–94%, Figure 4).

During high-temperature pyrolysis, the ordered glucose chains in crystalline cellulose dehydrate and cross-link into extended carbonaceous domains that progressively contract to form closed-pore walls. Concurrently, the amorphous hemicellulose and lignin fractions decompose into disordered, heteroatom-rich carbon matrices that impede excessive graphitization [23]. Tang et al. [23] demonstrated this closed-pore genesis mechanism using waste rosewood: precursors with high crystalline cellulose content (68.4%) yielded abundant closed-pore architectures (0.48 cm^3^ g^−1^) at 1500 °C, delivering a reversible capacity of 430 mAh g^−1^. The principal limitation of biomass is, however, its residual ash. Nita et al. [24] evaluated coconut shell, walnut shell, and corn silk, showing that elevated ash levels yield persistent inorganic impurities post-carbonization that act as catalytic loci for localized graphitization and exacerbate surface parasitic reactions, severely penalizing ICE. Comprehensive reviews by Thompson et al. [65] and Zhong et al. [66] consistently identify the spatial, temporal, and batch-to-batch variability of natural biomass as the primary bottleneck restricting its scalable industrial deployment.

Microstructurally, biomass-derived hard carbons exhibit a broad SSA distribution (<10 to 39 m^2^ g^−1^, Table 1), inherited from the vascular and cellular porosity of the precursor tissues. Escamilla-Pérez et al. [67] compared chitin and chitosan—structurally homologous yet compositionally distinct precursors—revealing that subtle variations in nitrogen-bearing functionalities dictate the post-carbonization mesostructure and ICE. To mitigate these natural variations, Xie et al. [22] achieved a remarkable ICE of 93.9% by molecular-level cross-linking to regulate biomass compositional ratios, proving the efficacy of compositional engineering prior to pyrolysis. Similarly, Li et al. [68] synergized cellulose enrichment with open-pore sealing, while Zhang et al. [25,69] pioneered data-driven and thermal-modulation strategies to optimize microstructural evolution. Complementary to these approaches, Minakshi and co-workers have pioneered the use of agricultural and industrial bio-wastes as carbon electrodes for Na-based energy devices: KOH activation of mango seed husk at 1100 °C yielded a specific surface area of 1943 m^2^ g^−1^ suitable for Na hybrid supercapacitors [70], while mild 450 °C activation of Australian hemp hurd produced a turbostratic honeycomb carbon delivering 240 F g^−1^ with well-defined D and G Raman signatures indicative of moderate defect density [71]. These works exemplify how biomass-specific activation thermodynamics can be tuned to target distinct Na-storage applications—from battery-type anodes requiring low SSA and high ICE to capacitive devices leveraging high SSA and heteroatom doping.

Within the proposed trade-off framework, biomass precursors are characterized by high and variable SSA (detrimental to ICE), substantial but cellulose-dependent closed-pore generation potential (conditionally favorable), and parasitic losses from inorganic ash. Maximizing the biomass ICE ceiling therefore requires a dual strategy, as follows: aggressive demineralization combined with selection or engineering of cellulose-rich feedstocks. A persistent unresolved question is whether ash-derived catalytic graphitization can ever be fully reconciled with closed-pore preservation, as follows: demineralization routes that effectively strip ash (e.g., HF/HCl washing) sometimes also disrupt the native cellular architecture that seeds closed porosity, and no consensus protocol currently balances these competing effects.

### 3.2. Synthetic Resin Precursors

Synthetic resins, predominantly phenolic resins, have attracted intense attention as premium hard carbon precursors owing to their moderate carbon yield (40–55%), tunable cross-linking density, and homogeneous molecular architectures [72]. Dey et al. [72] reviewed preparative strategies for phenolic-resin-derived hard carbons, identifying the density and topology of the three-dimensional cross-linked network as the primary lever for microstructural modulation.

The structural evolution of resin precursors is defined by early-stage thermosetting, as follows: phenolic monomers (e.g., phenol, resorcinol) undergo polycondensation with formaldehyde or alternative cross-linkers to form robust 3D networks. This extensive cross-linking sterically suppresses graphitic layer alignment during high-temperature carbonization, preserving a turbostratic configuration. Li et al. [26] used hexamethylenetetramine (HMTA) to cross-link a linear phenolic resin, achieving a near-zero SSA of 1.4 m^2^ g^−1^, closed pore volume of 0.315 cm^3^ g^−1^, ICE of 95.0%, and reversible capacity of 431 mAh g^−1^—the optimal ICE–capacity combination in this review (Figure 4). Conversely, Kamiyama et al. [27] carbonized a macroporous phenolic resin at 1500 °C, obtaining 386 mAh g^−1^ but ICE limited to ~80% due to elevated SSA (~24 m^2^ g^−1^), validating the inverse SSA–ICE correlation from Section 2.

Cross-linking density is the paramount variable governing the microstructural fate of resin derivatives. Guo et al. [28] engineered closed pores via hydrothermal cross-linking of resorcinol–benzaldehyde networks, while Zhao et al. [30] leveraged H_3_PO_4_-catalyzed cross-linking to achieve ICE of 89.7% at 1600 °C. Zhang et al. [29] introduced a pre-oxidation protocol to redirect the pyrolytic pathway of phenolic resins, and Wang et al. [73] advanced a pitch/polyacrylonitrile composite fiber architecture. Beda et al. [74] corroborated this cross-linking-centered design philosophy using green phloroglucinol–glyoxylic-acid resins, where cross-linking degree directly controlled the ultimate carbon yield (25–35%) and the resulting porosity, d_002_ and I_D/I_G. From an industrialization perspective, Zhu et al. [75] underscored that the mature synthetic chemistry and batch-to-batch consistency of phenolic resins constitute their most compelling commercial advantages.

Mapped onto the trade-off framework, resin-based precursors occupy a highly advantageous domain, as follows: intrinsically low SSA, robust closed-pore evolution, and moderate defect concentrations. The primary ICE-limiting factor is the generation of surface topological defects induced by CH_4_ and CO_2_ outgassing during intermediate pyrolysis; however, precise regulation of cross-linking density mitigates this outgassing-induced defect formation, establishing resins as the only precursor class to breach the 95% ICE threshold. A key reason resins consistently outperform pitch and coal on ICE—despite their lower carbon yield—lies precisely in this controllable outgassing regime. The moderate 40–55% carbon yield of resins reflects a measured release of volatiles that the rigid cross-linked network channels into inward pore closure rather than external micropore formation [72,75]. By contrast, the very high carbon yields of pitch (70–90%) and coal (80–95%) stem from already-condensed aromatic precursors that require little molecular reorganization during pyrolysis, leaving limited structural latitude to engineer turbostratic disorder and closed porosity. Conversely, the low yields of biomass and saccharides (10–25%) reflect violent gas release that preferentially generates open microporosity. Moderate, programmable outgassing—enabled by synthetic cross-linking—therefore emerges as the key thermochemical signature distinguishing resin-derived hard carbons.

### 3.3. Pitch-Based Precursors

Petroleum and coal tar pitches are complex mixtures of polycyclic aromatic hydrocarbons (PAHs), characterized by high H/C ratios and minimal heteroatom content. Their defining commercial attribute is an exceptional carbon yield of 70–90%. Yet this very attribute carries a thermodynamic penalty: the highly condensed aromatic nature of PAHs renders them susceptible to graphitization at elevated temperatures, and direct pyrolysis typically yields soft carbon or graphite rather than hard carbon [31].

Lu et al. [31] resolved this via an oxidative stabilization step (air pre-oxidation at 200–300 °C) prior to carbonization. The process introduces oxygen-bearing cross-linkages between PAH domains, sterically frustrating ordered molecular stacking and preserving a turbostratic structure during carbonization. Their optimized pre-oxidized petroleum pitch, carbonized at 1400 °C, delivered ICE of 88.6% and capacity of 300.6 mAh g^−1^. Zhang et al. [32] extended this approach by fractionating coal tar pitch, showing that the highly cross-linked tetrahydrofuran (THF)-insoluble fraction yields ICE of 90.96%—the highest value reported for pitch-derived systems.

The core mechanism for pitch-derived hard carbons hinges on the correlation between pre-oxidation degree and final microstructural topology. Investigations by Ji et al. [76], Li et al. [33] (MgO-catalyzed pre-oxidation), and Wu et al. [34] (molecular oxidative cross-linking) have established that the processing window for pitch stabilization is narrow, as follows: insufficient oxidation permits localized graphitization (contracting d_002_ and lowering ICE), while over-oxidation introduces excess surface oxygen functionalities that independently penalize ICE (Section 2.5).

Within the trade-off framework, pitch precursors possess a decisive advantage in SSA (typically <5 m^2^ g^−1^) and bulk structural homogeneity, which translates into the most tightly clustered ICE distribution among the five categories (86–91%, Figure 4). The ICE ceiling is nonetheless constrained by the narrow pre-oxidation window and by the intrinsic reluctance of pre-condensed PAH domains to develop closed porosity—the same high carbon yield that drives pitch’s cost advantage also limits its upside in the capacity–ICE plane (cf. Section 3.2). This trade-off notwithstanding, the combination of high carbon yield, cost-effectiveness, and low SSA has firmly established pitch as the dominant precursor in the rapidly scaling Chinese SIB industry.

### 3.4. Coal-Based Precursors

Coal (anthracite, sub-bituminous [77], and bituminous) is characterized by high fixed carbon, low volatile content, and highly condensed aromatic ring structures [78]. Kong et al. [78] and Liu et al. [79] reviewed the research status of coal-based hard carbons from macroscopic applications and recent advances, respectively. The causal trajectory for coal diverges fundamentally from the other four categories, as follows: because the pristine precursor already possesses a semi-ordered, graphitic-like short-range structure, thermal carbonization further drives graphitization rather than inducing the turbostratic disorder required for Na^+^ storage. In foundational work, Li et al. [36] directly pyrolyzed anthracite, obtaining a modest capacity of 222 mAh g^−1^ and ICE of 81%—values substantially inferior to competing precursor classes. Wang et al. [37] attempted to rectify this via alkaline demineralization (ash 9.38% → 1.00%); while capacity improved to 252 mAh g^−1^, ICE gains were marginal.

The suppressed ICE of coal-derived hard carbons stems from two intrinsic properties. First, mineral impurities (e.g., Si, Al, Fe) act as graphitization catalysts at high temperatures, generating electrochemically inert ordered domains that irreversibly trap sodium. Second, the pre-ordered molecular architecture of coal drives d_002_ toward severe contraction upon pyrolysis (0.355–0.370 nm, Table 1), presenting a thermodynamic and kinetic barrier to Na^+^ intercalation. To circumvent this, recent strategies pivot toward active pore generation: Wang et al. [38] used KOH activation to introduce closed pores into anthracite (ICE 82.3%, 308 mAh g^−1^); Huang et al. [39] and Zhang et al. [40] employed multi-effect pre-oxidation and citric acid activation, respectively, to disrupt the ordered domains.

Coal-based precursors therefore occupy the most challenging locus within the trade-off framework, as follows: over-contracted d_002_, moderate-to-high SSA from activation, mineral-induced parasitic losses, and an inherent resistance to closed-pore formation. The result is the lowest ICE tier (78–85%, Figure 4). The exceptionally high carbon yield of 80–95% that underpins coal’s cost advantage thus emerges, paradoxically, as the root of its structural limitation: the precursor carries almost no volatile inventory whose controlled release could drive turbostratic disorder or closed-pore genesis—a structural counterpart to the situation in pitch (Section 3.3) but at a more extreme end of the spectrum. For this reason, coal-derived hard carbons remain confined to cost-sensitive, grid-scale applications where absolute ICE maximization is a secondary consideration.

### 3.5. Saccharide and Simple Organic Precursors

Low-molecular-weight saccharides (e.g., glucose, sucrose) and simple organics serve as the model precursors in fundamental hard carbon research. They are distinguished by chemical purity, zero ash content, and isotropic molecular architectures. The foundational 2000 study by Stevens and Dahn [41] established the “intercalation–adsorption” dual-mechanism paradigm using glucose carbonized at 1000 °C.

The causal chain for saccharide-derived carbons follows a “pure yet highly porous” evolution. During intermediate pyrolysis, oxygen-rich saccharides undergo extensive dehydration and decarboxylation, releasing volatile gases (H_2_O, CO, CO_2_) that generate a highly developed open-pore network. Above 1400 °C, the localized growth of graphitic microcrystallites contracts the carbon matrix, converting a fraction of these open pores into functional closed pores. Kubota et al. [42] mapped this structural evolution for sucrose across 700–2000 °C, while Xiao et al. [43] achieved ICE of 86.1% and 361 mAh g^−1^ by kinetic control of the carbonization atmosphere to minimize outgassing-induced defects.

A hallmark of saccharide derivatives is their broad reversible capacity range (300–478 mAh g^−1^, the widest in Figure 4). To mitigate the open-porosity penalty, Li et al. [45] engineered “screening carbons” via chemical vapor deposition (CVD) of methane to conformally seal surface open pores, achieving ~390 mAh g^−1^. An alternative mechanistic route was established by Kamiyama et al. [44] through in situ MgO templating, whose operating principle warrants detailed discussion as it represents one of the most effective strategies for generating closed porosity in saccharide-derived hard carbon. In their protocol, magnesium gluconate is co-dissolved with glucose and freeze-dried to yield a homogeneous precursor blend. Upon pre-heating at 600 °C, the magnesium salt decomposes and Mg^2+^ ions migrate to form nanoscale MgO particles (5–20 nm) uniformly dispersed within the nascent carbon matrix. During subsequent high-temperature carbonization at 1500 °C, these rigid MgO nanoparticles act as sacrificial spacers: the surrounding carbon layers graphitize and densify around them while unable to collapse into the occupied voids. A final dilute-acid leaching step selectively dissolves the MgO, leaving behind size-defined, fully enclosed nanopores bounded by conformal graphitic walls. Crucially, because these engineered pores are sealed off from direct electrolyte contact, the massive capacity gain delivered by this approach (reversible capacity 478 mAh g^−1^—the highest in this review) is accompanied by ICE of 88%, decoupling the conventional SSA–capacity trade-off. The generality of this carbothermal-template mechanism has been recently extended by Igarashi et al. [80], who showed that ZnO and CaCO_3_ templates follow the same MO → M^0^ → gaseous-removal pathway; their ZnO-templated sucrose hard carbon achieved ICE of 91.7% at 464 mAh g^−1^, confirming that the mechanism is not MgO-specific but generalizable to any metal-oxide template whose carbothermal reduction product is volatile at the carbonization temperature. Further mechanistic refinements in pore architecture engineering and heteroatom doping have been advanced by Wu et al. [81], Yang et al. [82], and Mao et al. [83].

Within the trade-off framework, saccharide precursors occupy a paradoxical position. Although extreme thermal treatment can suppress SSA (<10 m^2^ g^−1^) and mitigate structural defects (I_D/I_G ~0.8) to give the “cleanest” carbon matrix among all categories, their ICE (77–88%) consistently underperforms optimized resins and pitches. Two persistent factors account for this, as follows: residual open microporosity that survives even aggressive thermal treatment, and the low ultimate carbon yield (15–25%) that inherently leaves a high density of topological edge defects. An unresolved debate in this subfield concerns whether template-free routes can ever match the ICE ceiling set by MgO/ZnO templating, as follows: recent studies consistently show that template-free saccharide hard carbons plateau near ICE of 87% even with optimized atmospheres and heating profiles, whereas templated systems exceed 88–92%, suggesting a mechanistic divide rather than a continuous optimization gradient. Consequently, saccharides remain invaluable as high-purity model systems for elucidating fundamental Na-storage mechanisms or achieving extreme theoretical capacities, but their poor mass economy and ICE ceiling preclude large-scale industrialization.

**Cross-Precursor Comparative Summary.** The ICE–capacity scatter matrix (Figure 4) shows that the five precursor families occupy distinct operational domains, as follows: pitches form a tight high-ICE cluster (86–91%, 301–345 mAh g^−1^); resins span a high-ICE, broad-capacity domain and define the absolute ICE ceiling (95%); coal-derived carbons occupy the low-ICE, low-capacity quadrant; saccharides display the broadest capacity variance at intermediate ICE; and biomass samples disperse across the entire ICE axis. These phenomenological divergences originate in each precursor’s distinct trajectory within the “SSA–closed pore–defect” trade-off space—resins achieve supremacy via simultaneous ultra-low SSA and extensive closed porosity, pitches dominate via extreme SSA suppression and bulk homogeneity, and coal systems are constrained by pre-ordered d_002_ contraction and catalytic mineral impurities.

The three-parameter framework also possesses predictive utility for emerging precursor candidates. Kraft lignin, combining moderate cross-linking density with low cellulose content and negligible ash after purification, is predicted to occupy an intermediate position between biomass and resin domains, with an ICE ceiling near 85–90% without post-treatment. Waste polyethylene terephthalate (PET), characterized by oxygen-rich backbones and zero ash, is predicted to follow a saccharide-like outgassing trajectory yielding high open porosity and moderate ICE (80–85%) unless mitigated by conformal coating. These predictions require experimental validation but illustrate how the framework can serve as a screening heuristic for unexplored precursor–process combinations.

## 4. Differentiated Regulation of ICE Through Carbonization Conditions and Post-Treatment Strategies Across Precursor Types

Section 3 systematically decoded the fundamental ICE heterogeneities arising from the distinct macromolecular compositions of the five precursor families. However, the ultimate ICE of a specific precursor can fluctuate by over 20 percentage points depending on the deployed carbonization protocols and post-treatment engineering. This underscores the paramount significance of process optimization. This section examines the differentiated cross-precursor effects of carbonization temperature, thermal atmosphere, and four primary categories of post-treatment strategies. By doing so, it establishes a rational, mechanistic basis for matching each precursor class with its optimal processing trajectory.

### 4.1. Differentiated Effects of Carbonization Temperature Across Precursor Types

Peak carbonization temperature is the primary thermochemical determinant of hard carbon microstructure, yet its impact diverges across precursor types in both trajectory and magnitude. Zheng et al. [84] mapped the structural evolution of sucrose-derived hard carbon from 800 to 1600 °C, observing monotonic ICE enhancement with temperature—consistent with the synergistic mechanism of Section 2 whereby elevated thermal treatment concurrently attenuates SSA, expands closed pore volume, and annihilates defects. Guo et al. [85] reported a broadly similar trend for biomass but with a lower optimal temperature (1400–1500 °C vs. 1500–1600 °C for saccharides), because inorganic ash in biomass catalyzes localized graphitization at extreme temperatures, sharply contracting d_002_ and penalizing Na^+^ intercalation.

Mohammed et al. [86] generalized this divergence into a simple heuristic, as follows: the higher the intrinsic molecular ordering of the pristine precursor, the lower its optimal carbonization temperature. This explains why pre-ordered coal reaches its relative ICE optimum at mild temperatures (~1200 °C, Li et al. [36]), whereas amorphous resins and saccharides require 1400–1600 °C to fully develop closed porosity. Ji et al. [87] further used machine learning to model these multivariable landscapes, showing that high-oxygen precursors (biomass) are far more sensitive to temperature modulation than low-oxygen networks (pitches)—supporting precision thermal protocol design.

Thermochemical optimization is therefore tied to precursor taxonomy, as follows: saccharides and resins require 1400–1600 °C, biomass optimizes at 1300–1500 °C, coal at 1200–1400 °C, and pitch occupies the narrowest window around 1400 °C due to pre-oxidation constraints. Two limitations of the reported literature should be acknowledged. First, the “optimal temperature windows” above are drawn from single-precursor studies, and no work to date has benchmarked these windows under a single unified experimental protocol across all five precursor categories—cross-study comparison therefore carries unavoidable confounding from electrolyte, current density, and electrode-preparation differences. Second, most temperature studies isolate temperature as a single variable, overlooking its coupling with heating rate and atmosphere (discussed in Section 4.2), which restricts the transferability of reported optima to industrial settings where all three parameters vary simultaneously.

### 4.2. Cross-Precursor Effects of Heating Rate and Atmosphere

Beyond peak temperature, thermal ramp kinetics and pyrolytic atmosphere exert profound—and strongly precursor-dependent—influences on microstructural evolution. Heating rate primarily controls the instantaneous flux of volatile release during intermediate pyrolysis. Xiao et al. [88] demonstrated, through multifield-regulated spark plasma sintering (heating rate 300–500 °C min^−1^, total synthesis < 1 min), that ultrafast heating effectively suppresses oxygen retention and defect density in biomass-derived hard carbon, delivering ICE of 88.9% and reversible capacity of 299.4 mAh g^−1^. The magnitude of this benefit, however, depends strongly on precursor volatile inventory, as follows: saccharide and biomass precursors (carbon yield 10–25%) are acutely sensitive to ramp kinetics because slow heating prolongs open-pore formation before subsequent closure is possible, whereas pitch and coal (carbon yield 70–95%) release so few volatiles that heating rate is largely inert. This precursor-dependent sensitivity has been largely overlooked in single-precursor heating-rate studies.

Atmosphere effects similarly show strong precursor dependence. Song et al. [89] introduced a mild-temperature H_2_/Ar reduction pretreatment (300–500 °C) for esterified starch-derived hard carbon, selectively stripping reactive C=O and COOH groups while preserving the carbon skeleton, thereby elevating ICE without sacrificing capacity. This reductive strategy benefits oxygen-rich precursors (biomass, saccharides) but is essentially inert for low-oxygen pitch and coal systems.

**Figure 5:**
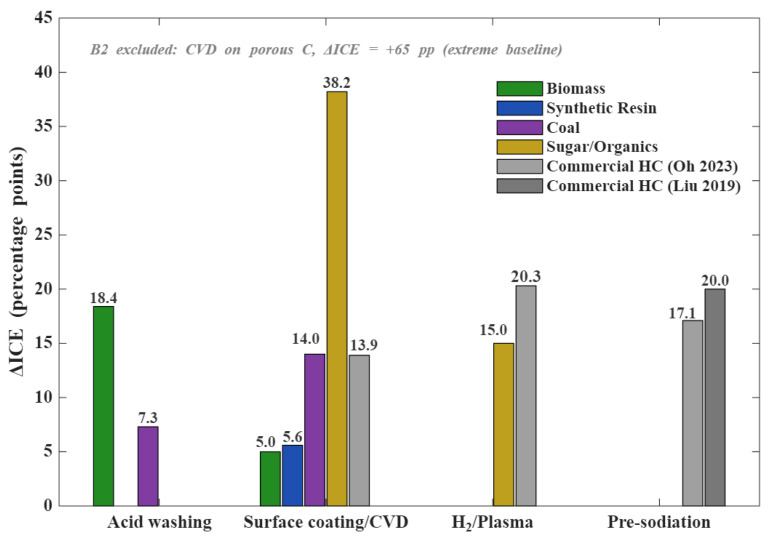
Comparison of ICE improvement (ΔICE) achieved by four post-treatment strategies across available precursor/material types. Commercial hard carbon data labeled as Liu 2019 and Oh 2023 correspond to Refs. [94] and [95], respectively.

In contrast, Zhang et al. [90] used N_2_ dielectric barrier discharge (DBD) plasma for non-thermal surface reconstruction, etching terminal oxygen groups and passivating defect sites while leaving d_002_ and closed pore volumes unaffected. This “surface-selective, bulk-preserving” paradigm is theoretically applicable to all precursors, but the achievable ΔICE (+10 to +20 percentage points, Figure 5) remains modest relative to coating or pre-sodiation strategies.

### 4.3. Precursor-Specific Applicability Matrix for Post-Treatment Strategies

Following primary carbonization, four post-treatment strategies—acid purification, conformal coating/CVD, reductive/plasma atmospheric treatment, and pre-sodiation—can be deployed to further elevate ICE. As shown in Figure 5, the ΔICE efficacy of these interventions is not universal but exhibits a highly precursor-specific response profile.

Acid washing is the most facile purification strategy, using aqueous HCl or HF to dissolve pyrolytic ash. It yields ΔICE of +7 to +18 percentage points (Figure 5), but its utility is governed by initial ash content: transformative for high-ash biomass (shell and husk derivatives), redundant for pure resins and pitches, and moderately beneficial for coal. This corroborates the Section 3 conclusion that inorganic ash is the primary ICE bottleneck for biomass.

Surface coating and chemical vapor deposition (CVD) enhance ICE by conformally sealing open micropores, suppressing the electrochemically active SSA. Liu et al. [91] coated a phenolic resin core with a pitch-derived soft carbon shell, raising ICE from 82.6% to 88.2% (ΔICE +5.6). Pan et al. [92] refined this through kinetically controlled micropore infiltration; Trinh et al. [93] used plasma-enhanced CVD for graphitic passivation of binder-free architectures. Coating offers the widest ΔICE spectrum (+5 to +38, Figure 5), with magnitude correlating directly to initial SSA and open porosity, as follows: high-SSA precursors (biomass, low-temperature saccharides) benefit massively, while dense low-SSA pitch carbons offer minimal driving force for further surface sealing.

Pre-sodiation establishes the absolute ΔICE ceiling (+17 to +20 percentage points, Figure 5). Rather than modifying intrinsic microstructure, it preempts first-cycle losses by artificially loading the anode with sodium prior to full-cell assembly. Methodologies range from chemical infusion (sodium naphthalenide/THF, Liu et al. [94]) to solid-state thermal reduction (NaBH_4_ decomposition, Oh et al. [95]). Because it circumvents structural engineering, pre-sodiation is universally applicable across precursor classes, although stringent atmospheric requirements and manufacturing complexity currently hinder commercial adoption. Parallel innovations in nanostructural engineering (He et al. [96]) and waste valorization (sorghum distillers’ grains, Ao et al. [97]) further expand the post-treatment toolbox.

Post-treatment deployment must therefore be mapped to each precursor’s structural deficit, as follows: biomass requires acid washing (ash) and heavy carbon coating (porosity); resins and pitches benefit from mild vapor-phase passivation (defects); coal demands sequential demineralization and pore-sealing; saccharides require robust CVD coating for open-pore mitigation. When intrinsic interventions fail to meet the commercial ICE threshold, pre-sodiation remains the extrinsic compensatory option. This precursor–strategy matching rationale underpins the decision roadmap in Section 5.

### 4.4. Electrolyte Effects on ICE Across Precursor Types

Electrolyte chemistry constitutes a fourth regulatory axis that intersects with the three structural parameters discussed above, yet it is often treated as a fixed background variable in precursor-focused studies. The manuscript dataset itself reveals the magnitude of this effect: waste rosewood hard carbon tested in a NaPF_6_/DME ether electrolyte ([23], Table 1) delivers 430 mAh g^−1^ with reversible behavior distinctly superior to morphologically comparable biomass carbons tested in carbonate systems [24,25].

The origin of this divergence lies in the distinct solid electrolyte interphase (SEI) formed in ether versus carbonate electrolytes. Zhang et al. [98] established that diglyme-based electrolytes produce a thinner, inorganic-rich, and chemically more stable SEI than EC/DEC systems, directly translating into higher first-cycle reversibility on disordered carbons. Li et al. [99] subsequently demonstrated using in situ Raman, XRD, and EIS that the charge-transfer activation barrier at the electrolyte–carbon interface is markedly lower in ether systems, attributed to the weakly coordinating Na^+^-diglyme solvation shell that enables near-barrier-free desodiation. Hirsh et al. [100] further quantified that the thick, organic-rich SEI generated in carbonate electrolytes is the primary source of low ICE and accelerated capacity fade in hard carbon anodes.

Importantly, the magnitude of the ether benefit depends on precursor microstructure. High-SSA, high-defect hard carbons (biomass, low-temperature saccharides) reap the greatest ICE uplift because the SEI-forming area is large; dense, low-SSA pitch-derived carbons benefit less because their SEI formation is already minimized. This coupling means that precursor selection and electrolyte selection should be co-optimized rather than treated independently—a perspective largely absent from the current literature, where most precursor-optimization studies fix the electrolyte a priori, making it difficult to deconvolute precursor effects from electrolyte effects. Standardized reporting of electrolyte composition alongside structural parameters would substantially strengthen cross-study comparability, an issue revisited in the Outlook.

## 5. Precursor Selection Roadmap Oriented Toward Full-Cell Applications

While the preceding sections systematically deconstructed hard carbon ICE across three complementary dimensions—microstructural correlative frameworks (Section 2), precursor-specific causal continua (Section 3), and thermochemical process optimization (Section 4)—these analyses were fundamentally anchored in half-cell test paradigms. In practical full-cell architectures, the detrimental impact of a low ICE is profoundly amplified. Because the total active sodium inventory is intrinsically finite and exclusively cathode-derived, every stoichiometric unit of irreversible sodium consumption at the anode directly penalizes the deliverable energy density of the full cell. This section first quantifies the deterministic influence of ICE on critical full-cell performance metrics, and subsequently integrates the analytical outcomes of the preceding sections to construct a precursor selection decision roadmap tailored for pragmatic techno-economic scenarios.

### 5.1. Quantitative Impact of ICE on Full-Cell Performance Metrics

The transition from half-cell to full-cell testing fundamentally redefines the significance of ICE. In a half-cell, the metallic sodium counter electrode acts as a virtually infinite reservoir; anodic irreversible losses merely suppress the calculated ICE. In a full cell, however, sodium ions sequestered during the first cycle are withdrawn from the finite cathode inventory and cannot be replenished. Chen et al. [101] quantified this penalty as follows: raising hard-carbon ICE from 75% to 90% yields ~20% more first-cycle reversible capacity in the full cell, as a larger fraction of the cathode inventory remains electrochemically active.

Pei et al. [102] re-evaluated anode research through a full-cell lens, showing that low ICE triggers a cascading degradation chain as follows: excessive first-cycle SEI → accelerated electrolyte consumption → premature depletion → shortened cycle life. Lu et al. [103] addressed this via ion desolvation modulation as follows: an extended aging protocol drives the nanopore-confined electrolyte into aggregated solvation configurations, suppressing reductive decomposition and achieving an average Coulombic efficiency of 98.21%.

Fan et al. [104] highlighted the coupling between cathode–anode capacity matching (N/P ratio) and anodic ICE: when anode ICE falls below 85%, stable cycling typically requires an N/P ratio > 1.2, i.e., >20% excess anodic active material, degrading energy density and raising cell costs. Zheng et al. [105] validated this in poplar-derived hard carbon full cells, confirming the techno-economic benefits of high-ICE (>85%) architectures for minimizing N/P.

The pragmatic ICE prerequisites for full-cell deployment can therefore be stratified into three tiers, as follows: (i) Baseline (ICE ≥ 80%), sufficient for cost-sensitive grid storage with elevated N/P ratios as compensation; (ii) Industrial (ICE ≥ 90%), meeting mainstream commercial demands (power batteries, consumer electronics) with N/P constrained to 1.05–1.10; and (iii) Premium (ICE ≥ 92%), enabling next-generation high-energy-density cells with N/P approaching 1.0. These three thresholds form the branching criteria of the decision roadmap below.

### 5.2. Precursor Selection Decision Roadmap

**Figure 6:**
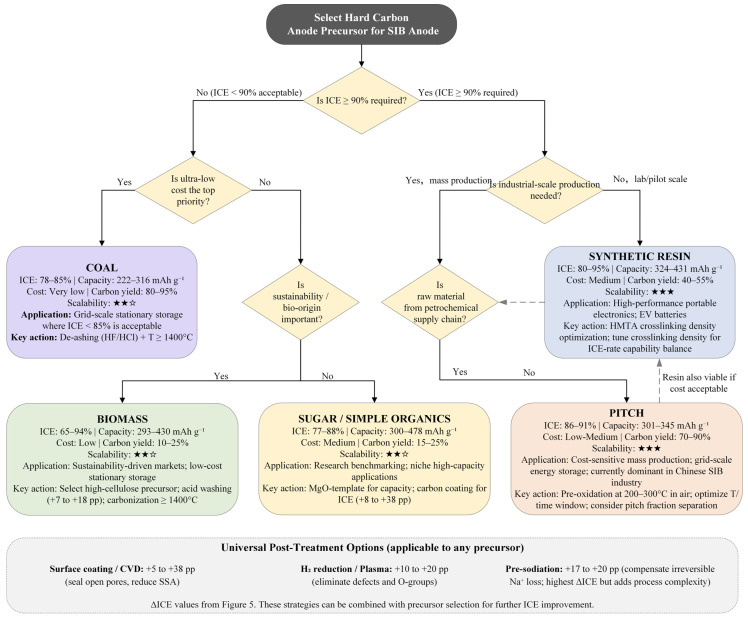
Decision tree for hard carbon precursor selection based on application requirements.★★★ indicates industrial-scale readiness, whereas ★★☆ indicates pilot-scale demonstration.

Building on the causal chain analyses (Section 3), process optimization (Section 4), and the ICE threshold hierarchy above, we construct a precursor selection roadmap for scalable industrial deployment (Figure 6), supported by a cross-categorical synthesis matrix (Table 2).

The primary node of the decision tree bifurcates by target ICE. When an application tolerates ICE < 90% (Baseline Tier), economics and sustainability dominate. If ultra-low cost is the primary objective, coal-derived precursors are preferred for their raw-material affordability and high carbon yield (80–95%, Table 2); the intrinsic ICE deficit (78–85%) can be offset by elevated N/P ratios in stationary storage. Where ecological sustainability is prioritized, biomass precursors prevail, provided ICE is raised above 80% through acid demineralization (ΔICE +7 to +18, Figure 5) and cellulose-rich feedstock selection. When neither cost nor scale drives the decision, saccharides serve as research-grade or niche-capacity models, with MgO-templating delivering up to 478 mAh g^−1^.

When ICE ≥ 90% is mandated (Industrial and Premium Tiers), scalability and microstructural consistency become the branching criteria. Although Wang et al. [106] showed that full-cell pre-sodiation can artificially elevate low-ICE precursors, the added process complexity and cost currently limit its industrial reach. For massive-scale production, pitch-based precursors offer the most mature techno-economic pathway, combining high carbon yield (70–90%), stable petrochemical supply chains, and an ICE ceiling of ~91% via pre-oxidation—this is why pitch currently dominates the rapidly scaling Chinese SIB industry [107]. For pilot-scale or premium applications, synthetic resins (particularly HMTA-crosslinked phenolic networks) remain the only category consistently breaching 95% ICE, with programmable macromolecular architectures and batch-to-batch consistency making them indispensable for high-end consumer and motive power applications.

Precursor selection cannot be fully decoupled from kinetic considerations. Xiao et al. [108] showed that closed-pore dimensions strongly influence fast-charging capability, requiring precursor screening to balance thermodynamic ICE targets with rate-capability requirements. Wang et al. [109] further demonstrated the efficacy of core–shell composite architectures as a spatial design dimension of precursor engineering.

The decision roadmap (Figure 6) is not a rigid framework but a flexible baseline onto which extrinsic post-treatment strategies can be superimposed. As mapped in Figure 5, conformal coating (+5 to +38 pp), atmospheric plasma/reduction (+10 to +20 pp), and pre-sodiation (+17 to +20 pp) act as universal compensatory levers. A low-cost biomass precursor initially limited to 65% ICE, for example, can legitimately enter the Industrial Tier (>90%) through a sequence of acid washing and CVD coating. This synergistic paradigm—intrinsic precursor selection combined with extrinsic post-treatment stacking—is the blueprint for translating laboratory hard-carbon innovations into commercial SIB technologies. The cumulative effects of stacking multiple post-treatments remain underexplored, presenting a frontier for future quantitative work.

## 6. Conclusions and Outlook

This review demonstrates that the precursor dependence of hard carbon ICE is not governed by any single structural variable but emerges as the terminal output of a causal continuum spanning chemical composition → carbonization → microstructure → electrochemical response. Through the “SSA–closed pore–defect” three-parameter trade-off framework, we show that each precursor family occupies a distinct and predictable position within this space, which dictates its ICE range and optimization ceiling. By transcending single-precursor and isolated-strategy paradigms, this work provides the first unified cross-categorical benchmarking of five major precursor classes and a quantitative precursor selection decision roadmap oriented toward practical full-cell deployment.

The framework furnishes actionable techno-economic guidance, as follows: pitch is the most industrially mature pathway for ICE ≥ 90% at scale; synthetic resins remain the sole category consistently breaching 95% ICE; and biomass and coal systems, though constrained by intrinsic compositional limitations, can be elevated through targeted post-treatment stacking to meet baseline requirements.

Despite these contributions, three frontiers warrant focused investigation. First, the cumulative effects of combinatorial post-treatments (e.g., acid washing coupled with carbon coating or pre-sodiation) remain uncharted; orthogonal combinations targeting distinct ICE loss mechanisms may surpass the ceiling of any individual intervention. Second, machine-learning-assisted optimization of the high-dimensional “precursor × carbonization × post-treatment” parameter space offers a promising route to accelerating the discovery of optimal precursor–process combinations. Third, standardized electrochemical testing protocols—unified electrolyte formulations (especially critical given the carbonate-versus-ether ICE divergence documented in Section 4.4), voltage windows, current densities, and electrode mass loadings—are essential for genuinely comparable cross-study ICE data. Only on such a foundation can the comparative framework evolve into a continuously refined commercialization guide.

## Figures and Tables

**Table 1:** Summary of representative structural parameters and electrochemical performance of hard carbons derived from five precursor categories.

No.	Precursor	Category	T (°C)	SSA (m^2^ g^−1^)	d_002_(nm)	V_Closed (cm^3^ g^−1^)	I_D/I_G	ICE (%)	Capacity (mAh g^−1^)	Electrolyte	Ref.
1	Bamboo + maleic anhydride crosslinked	Biomass	1500	2.37	0.384	0.475	1.08	93.9	324	NaPF_6_/EC:DEC	[22]
2	Waste rosewood (H-1500)	Biomass	1500	2.6	~0.370	0.48	1.295	NR	430	NaPF_6_/DME ‡	[23]
3	Walnut shell	Biomass	1300	<10	0.387	NR	1.02	~72	315	NaClO_4_/EC:PC	[24]
4	Coconut shell	Biomass	1300	8.2	0.390	NR	1.05	~68	293	NaClO_4_/EC:PC	[24]
5	Red yeast rice	Biomass	1200	38.9	0.389	NR	NR	~65	322.6	NaPF_6_/EC:DEC	[25]
6	Phenolic resin + HMTA (CPF-1400)	Resin	1400	1.4	0.381	0.315	NR	95.0	431	NaPF_6_/ester	[26]
7	Macroporous phenolic resin	Resin	1500	~24	0.370	NR	NR	~80	386	NaPF_6_/carbonate	[27]
8	HMTA-crosslinked linear phenolic resin	Resin	1400	NR	NR	NR	NR	90.7	323.9	NaPF_6_/carbonate	[28]
9	Pre-oxidized phenolic resin	Resin	1400	~8	0.382	NR	~1.0	84.7	334.3	NaClO_4_/EC:DEC	[29]
10	Crosslinked phenolic resin + H_3_PO_4_	Resin	1600	NR	~0.375	NR	~1.0	89.7	416.1	ester-based	[30]
11	Petroleum pitch (pre-oxidized 300 °C)	Pitch	1400	~3.5	~0.388	NR	NR	88.6	300.6	NaClO_4_/EC:PC	[31]
12	Coal tar pitch (THF-insoluble fraction)	Pitch	1400	NR	0.380	NR	~0.85	90.96	309.7	NaPF_6_/carbonate	[32]
13	Pitch + MgO catalytic preoxidation	Pitch	1400	NR	~0.385	NR	NR	88.5	321.7	NaPF_6_/carbonate	[33]
14	Pitch + calcium gluconate crosslinker	Pitch	1400	~5	~0.383	NR	NR	~87	345.4	NaPF_6_/carbonate	[34]
15	Pitch + NH_4_H_2_PO_4_ pre-oxidation	Pitch	1400	NR	0.389	NR	NR	~86	340	carbonate	[35]
16	Pyrolyzed anthracite	Coal	1200	NR	~0.360	NR	NR	81.0	222	carbonate	[36]
17	Deashed anthracite (alkali-purified)	Coal	1400	~12	~0.355	NR	NR	~78	252	NaClO_4_/EC:PC	[37]
18	Anthracite + KOH activation	Coal	1400	NR	~0.365	NR	NR	82.3	308	carbonate	[38]
19	Bituminous coal (preoxidized)	Coal	1400	~15	~0.370	NR	~1.1	~85	~290	NaPF_6_/carbonate	[39]
20	Bituminous coal + citric acid	Coal	1300	NR	NR	NR	NR	~82	315.5	NaPF_6_/carbonate	[40]
21	Glucose (Stevens & Dahn, classic)	Sugar	1000	NR	~0.380	NR	NR	~77	~300	NaClO_4_/EC; 0–1.2 V	[41]
22	Sucrose (HC-1500)	Sugar	1500	<10	0.374	NR	NR	~87	~330	NaPF_6_/carbonate	[42]
23	Sucrose (low-defect, low-porosity)	Sugar	1600	~3	~0.380	NR	~0.8	86.1	361	NaClO_4_/EC:DEC	[43]
24	Glucose + Mg gluconate (MgO-template)	Sugar	1500	NR	0.376	NR	NR	88.0	478	NaPF_6_/PC	[44]
25	Sucrose sieving carbon (CVD modified)	Sugar	900 (CVD)	<0.5	NR	NR	NR	~80	~390	NaPF_6_/carbonate; 0.005–2.5 V	[45]

NR = not reported. ~ = approximate. ‡ DME electrolyte. Chem. Eng. J. 2024. See Section 3.2. BET SSA ≈ 0. All data at 20–50 mA g^−1^, 0–2.0 V unless noted. Ref. [41] uses 0–1.2 V; Ref. [45] uses 0.005–2.5 V.

**Table 2:** Comprehensive comparison of five hard carbon precursor categories for sodium-ion battery anodes.

Parameter	Biomass	Synthetic Resin	Pitch	Coal	Sugar/Simple Organics
ICE range (%)	65–94	80–95	86–91	78–85	77–88
Reversible capacity (mAh g^−1^)	293–430	324–431	301–345	222–316	300–478
Typical SSA (m^2^ g^−1^)	<10–39	1–24	3–5	12–15	<10
d_002_ range (nm)	0.370–0.390	0.370–0.382	0.380–0.389	0.355–0.370	0.374–0.380
Carbon yield (%)	10–25	40–55	70–90	80–95	15–25
Raw material cost	Low	Medium	Low–Medium	Very Low	Medium
Supply stability	Seasonal/Regional	Stable (industrial)	Stable (petrochemical)	Abundant (mining)	Stable (industrial)
Batch consistency	Poor	Excellent	Good	Moderate	Good–Excellent
Scalability	★★☆	★★★	★★★	★★☆	★★☆
Key ICE-limiting factor	High SSA from intrinsic porosity; high ash content	Surface defects from CH_4_/CO_2_ release during pyrolysis	Requires pre-oxidation to prevent graphitization; narrow window	Mineral impurities; low d_002_ from ordered precursor	High open porosity at low T; low carbon yield (~15–25%)
Most effective ICE strategy	Acid washing (+7 to +18 pp); closed-pore engineering	Crosslinking regulation; soft-carbon coating (+5–6 pp)	Pre-oxidation T/time optimization; fraction separation	De-ashing + high-T carbonization; surface coating (+14 pp)	MgO-template for capacity; carbon coating (+8 to +38 pp)
Full-cell readiness	Medium (prototype cells)	High (18,650 cylindrical cells)	High (industrial production in China)	Medium–High (cost advantage for grid)	Medium (lab-scale; high cost barrier)
Recommended application	Low-cost stationary storage; sustainability-driven	High-performance portable electronics; EV	Cost-sensitive mass production; grid-scale	Ultra-low-cost grid storage (ICE < 85% acceptable)	Research benchmarking; niche high-capacity

Scalability: ★★★ = industrial scale; ★★☆ = pilot scale demonstrated. pp = percentage points. ICE and capacity ranges based on Table 1 (25 representative studies). Carbon yield = mass ratio of HC product to precursor at 1200–1500 °C. Full-cell readiness assessed from published full-cell or prototype demonstrations. ΔICE values from Figure 5 dataset.

## Data Availability

No new data were created or analyzed in this study. Data sharing is not applicable to this article.
